# Comparative genomic analysis of the multispecies probiotic-marketed product VSL#3

**DOI:** 10.1371/journal.pone.0192452

**Published:** 2018-02-16

**Authors:** François P. Douillard, Diego Mora, Robyn T. Eijlander, Michiel Wels, Willem M. de Vos

**Affiliations:** 1 Research Program Unit Immunobiology, Medicum, University of Helsinki, Helsinki, Finland; 2 Department of Food Hygiene and Environmental Health, Faculty of Veterinary Medicine, University of Helsinki, Helsinki, Finland; 3 Department of Food, Environmental, and Nutritional Sciences (DeFENS), University of Milan, Milan, Italy; 4 NIZO, Ede, The Netherlands; 5 Laboratory of Microbiology, Wageningen University, Wageningen, The Netherlands; University of Torino, ITALY

## Abstract

Several probiotic-marketed formulations available for the consumers contain live lactic acid bacteria and/or bifidobacteria. The multispecies product commercialized as VSL#3 has been used for treating various gastro-intestinal disorders. However, like many other products, the bacterial strains present in VSL#3 have only been characterized to a limited extent and their efficacy as well as their predicted mode of action remain unclear, preventing further applications or comparative studies. In this work, the genomes of all eight bacterial strains present in VSL#3 were sequenced and characterized, to advance insights into the possible mode of action of this product and also to serve as a basis for future work and trials. Phylogenetic and genomic data analysis allowed us to identify the 7 species present in the VSL#3 product as specified by the manufacturer. The 8 strains present belong to the species *Streptococcus thermophilus*, *Lactobacillus acidophilus*, *Lactobacillus paracasei*, *Lactobacillus plantarum*, *Lactobacillus helveticus*, *Bifidobacterium breve* and *B*. *animalis* subsp. *lactis* (two distinct strains). Comparative genomics revealed that the draft genomes of the *S*. *thermophilus* and *L*. *helveticus* strains were predicted to encode most of the defence systems such as restriction modification and CRISPR-Cas systems. Genes associated with a variety of potential probiotic functions were also identified. Thus, in the three *Bifidobacterium* spp., gene clusters were predicted to encode tight adherence pili, known to promote bacteria-host interaction and intestinal barrier integrity, and to impact host cell development. Various repertoires of putative signalling proteins were predicted to be encoded by the genomes of the *Lactobacillus* spp., *i*.*e*. surface layer proteins, LPXTG-containing proteins, or sortase-dependent pili that may interact with the intestinal mucosa and dendritic cells. Taken altogether, the individual genomic characterization of the strains present in the VSL#3 product confirmed the product specifications, determined its coding capacity as well as identified potential probiotic functions.

## Introduction

There has been a steady interest in functional food products consisting of live lactic acid bacteria (LAB) and bifidobacteria that are marketed as probiotics, defined as ‘live microorganisms that, when administered in adequate amounts, confer a health benefit on the host’ [1]. The majority of these products consist of single strains of LAB or bifidobacteria, and have been supported by research with a varying level of sophistication. In some cases, probiotic-marketed products are poorly characterized and insight in their mechanisms of action is lacking, based on only animal studies, or on human studies without appropriate controls. This impedes further applications in improving the quality of life or treating diseases. Moreover, the absence of appropriate characterization limits comparative analysis and prevents predicting the product efficacy.

The limited knowledge of many marketed probiotics contrasts with the fact that several strains of *Lactobacillus* and *Bifidobacterium* spp. have been extensively characterized and used in numerous well-performed trials. When the number of publications is taken as an indicator, *Lactobacillus rhamnosus* GG is the most studied strain marketed as a probiotic. The *L*. *rhamnosus* GG genome has been characterized and used for comparative genomics studies with other isolates, resulting in the identification of mucus-binding sortase-dependent pili [2–4]. These long protruding protein polymers (pili) not only bind to the host mucosa and outcompete potential pathogens, but also affect immune stimulation via interaction with dendritic cells [5,6]. Similarly, another LAB, *L*. *acidophilus* NCFM has been well-characterized for its genome and functional properties, including the S-layer protein and associated proteins that have been found to interact with the DC-SIGN receptor of dendritic cells [7–9]. *L*. *plantarum* WCFS1 has been the first *Lactobacillus* strain to be genomically characterized and served as paradigm for several studies aiming to understand the interaction with the host [10–13]. The widely marketed strain *Bifidobacterium animalis* subsp. *lactis* BB-12 has also been genomically characterized and compared to other *Bifidobacterium* species [14–16]. A specific set of Tad pili and sortase-dependent pili has been discovered in *B*. *breve* that are involved in intestinal persistence and the host-microbe dialogue [17,18]. Surface exopolysaccharides in *B*. *breve* UCC2003 were also shown to be involved in host-microbe cross-talk [19].

Genomic characterizations have been instrumental in discovering the molecular basis of the host-interaction of industrial strains as well as providing an overview of their future capacities [20,21]. Moreover, genome-based analysis has been used for investigating the stability of industrial strains in the laboratory and products [22]. This approach has shown that some widely commercialized strains of *L*. *casei* or *B*. *animalis* share a recent common ancestor [15,23]. These studies were all performed with single strains but several products also contain multiple strains of lactic acid bacteria or bifidobacteria. This includes the multispecies product VSL#3 that is used for treating various gastro-intestinal disorders, such as ulcerative colitis, pouchitis, and irritable bowel syndrome [24–26]. Several trials have shown the effectiveness of VSL#3 that originally was reported to include bacteria of the species *Streptococcus salivarius* subsp. *thermophilus* (now known as *S*. *thermophilus*), *L*. *acidophilus*, *L*. *casei*, *L*. *plantarum*, *L*. *helveticus* (first described as *L*. *bulgaricus* subsp. *delbrueckii)*, and three strains of bifidobacterial species [27,28]. Based on 16S rRNA sequences, the bifidobacterial strains were suggested to belong to *B*. *breve*, *B*. *longum* and *B*. *infantis* [29]. However, in the meantime *B*. *longum* and *B*. *infantis* have reported to be a single species and mislabelling of bifidobacteria in commercial products has been reported to occur often [30]. Genome-based approaches can be used to identify bacterial strains notably by comparison with genomically characterized type strains, which is highly relevant for regulatory and scientific purposes [31].

In contrast to conventional marketed products that are consumed in a daily dose of approximately 10^9^ bacterial cells per dose, the multispecies VSL#3 product is typically used in treatments at doses of 450–900 10^9^ cells per day. Significant improvement by VSL#3 of disease symptoms were observed in the treatment of ulcerative colitis and pouchitis, an inflammation of the ileal pouch in colectomy patients [24,32]. Moreover, the VSL#3 product was found to reduce the disease severity and hospitalization period of patients with liver cirrhosis and hepatic encephalopathy, improved non-alcoholic fatty liver disease in obese children possibly *via* increasing GLP-1 production, and correcting the inflammatory status of obese adults [33–35]. These clinical trials have been paralleled by a variety of *in vitro* studies that supported the therapeutic effects and have shown the capacity of the VSL#3 product to affect the immune function associated with specific transcriptional response [36–38]. However, the effector molecules produced by VSL#3 that could contribute to these effects have not been identified yet. To further comprehend the mode of action of VSL#3 and generate a basis for future work, the genomes of all 8 bacterial strains present in this multispecies product were sequenced, used to assess their taxonomic position and predict their function, and compared to other well-characterized single industrial bacterial strains.

## Materials and methods

### Bacterial strains and growth conditions

All eight bacterial strains that make up the VSL#3 product were individually provided by the manufacturer (courtesy of Actial Farmaceutical SRL, Rome, Italy), coded and cultivated as listed in S1 Table. Five ml of overnight-grown cultures were then further used for genomic DNA isolation. Cells were lyzed using lysozyme (20mg/ml), mutanolysin (10U/ml), 1% w/v SDS, 50 μg/ml RNase and 300 μg/ml proteinase K followed by an incubation of 30 min at 37 ^o^C. Genomic DNA was then isolated from cell lysates with a RSC Blood DNA kit AS1400 according to the manufacturer’s protocol on a Promega Maxwell RCS instrument (Promega, Madison, WI, USA). DNA was quantified using Quanti-iT™ Pico Green dsDNA Assay (Invitrogen, San Diego, CA, USA).

### Evaluation of minimal inhibitory concentration (MIC) of antibiotics against VSL#3 strains

Minimum inhibitory concentration (MIC) was determined using the standard microdilution method for drug susceptibility testing [39,40]. Data are reported as the average of two independent assays. The MICs were evaluated in LSMa (ISO-Sensitest broth, Oxoid supplemented with 10% v/v M17 Difco) for *S*. *thermophilus*, in LSMb (ISO-Sensitest broth, Oxoid supplemented with 10% v/v MRS Difco) for lactobacilli, and in LSMc (ISO-Sensitest broth, Oxoid supplemented with 10% v/v MRS Difco supplemented with 0.05% L-Cys) for bifidobacteria. The reference strain *L*. *paracasei* LMG12586 (ATCC334) reported in the ISO10932/IDF233 2010 document [41] was included for comparisons and interlaboratory range analysis.

### Genome sequencing, assembly and annotation

Purified genomic DNA was sent for paired end (150 nt, insert of 300 bp) whole genome sequencing using the next-generation sequencing platform (HiSeq2500, Illumina) at BaseClear (The Netherlands) with an expected coverage of 150x and an output of approximatively 100 contigs. The quality of the FASTQ sequences was enhanced by trimming off low-quality bases using the “Trim sequences” option of the CLC Genomics Workbench version 9.5.1. The quality filtered sequence reads were assembled to contigs using the “De novo assembly” option of the CLC Genomics Workbench version 9.5.1 (Qiagen). Mis-assemblies and nucleotide disagreement between the Illumina data and the contig sequences were corrected with Pilon version 1.20 [42]. Subsequently, the contigs were linked and placed into scaffolds, where the orientation, order and distance between them were estimated using the insert size between the paired-end reads. The analysis was performed using the SSPACE Premium Scaffolder version 2.3 [43]. The gapped regions within the scaffolds were (partially) closed in an automated manner using GapFiller version 1.10 [44]. Genome annotation was then performed on the assembled contig or scaffold sequences using the BaseClear annotation pipeline, which is based on the Prokka Prokaryotic Genome Annotation System (version 1.6) [45]. The pipeline includes a number of features, including Prokaryote gene prediction by Prodigal v2 [46], rRNA using barrnap v0.2 (Victorian Bioinformatics Consortium, http://www.vicbioinformatics.com/software.barrnap.shtml), tRNA prediction by Aragorn v1.2.36 [47], and pCDS physical-chemical properties using an in-house script. On the inferred proteins, the following downstream analyses were performed: prediction of EC number, CAZY number and function annotation from UniProt BLAST best hit, Signal peptide prediction and cellular localization using SignalP v4 [48], and conserved domains by hmmer-3 [49].

### Species assignment and phylogenetic analysis

Two different methods were used to determine the species of the sequenced strains. First, 16S rRNA gene sequences were compared using the blast option of the greengenes database (http://greengenes.lbl.gov/) [50]. Secondly, proteome comparisons were performed by a BLASTp of a random set of 500 protein sequences of each genome against the collection of reference and representative genomes from the NCBI genome database (February 2017). BLASTp was run using standard settings, picking the hit with the lowest e-value for each of the 500 proteins (best hit), where a random subset of 500 proteins was searched against the refseq database of complete genomes. Species calling was determined based on the species with best hits among the 500 protein sequences.

For the phylogenetic (core genome) tree, the genomes of the strains from the product were compared to a set of relevant reference and representative genomes (NCBI genome database sept 25th 2017) using OrthoMCL [51]. The amino acids differences in the core (conserved) proteome set were aligned and a tree was generated as previously described [52].

### Bioinformatic analyses

The annotated genome sequence of all eight bacterial strains were analyzed for the presence of antibiotic resistance genes using the resfams database [53] and was performed with HMM-er3 [49] using the HMMs provided by resfams and the thresholds for detection as stated by resfams and provided within the HMM. The protein sequences were compared with a recently downloaded version (November 2016) of the Virulence Data base (VFDB) [54] using blast [55]. The output results were subsequently filtered for relevance based on the following criteria (E-value < 0.01; >50% amino acid sequence identity; >250 alignment length). The CRISPR-Cas loci were first identified based on the initial genome prediction and further analyzed using CRISPRFinder [56], CRISPR Target [57] and BLAST analysis [55] adjusted for short query sequences. Mobilome genes, *i*.*e*. insertion elements, transposases and phages, were detected using keyword searches against the genome annotation. LPXTG proteins were initially identified using the method as previously described by Boekhorst and colleagues [58], followed by an additional verification using CW-PRED [59], to remove any possible false-positive or ambiguous hits.

### Genome sequence accession numbers

The genome sequences of all eight strains present in the multispecies product VSL#3 were deposited in NCBI's Sequence Read Archive (SRA) under the following biosample accession numbers (SAMN07187782, SAMN07187783, SAMN07187784, SAMN07187785, SAMN07187786, SAMN07187787, SAMN07187788 and SAMN07187789) and under the following genome accession numbers (NIGX00000000, NIGW00000000, NIGV00000000, NIGU00000000, NIGT00000000, NIGS00000000, NIGR00000000 and NIGQ00000000).

## Results and discussion

### Overview of genome sequences and species classification

Total DNA of the eight VSL#3 strains, which were obtained from the manufacturer, was isolated and used to obtain draft genome sequences that had a coverage ranging from 264x to 462x with a relatively low amount of scaffolds (in between 13–47), except for *Lactobacillus helveticus* BT08, which had 132 scaffolds possibly due to a large number of transposons in the genome (S3 Table). The strains were taxonomically assigned to a species based on their 16S rRNA gene sequences and this analysis identified 6 out of 8 strains (BT01, BA05, BP06, BP07, BL03 and BI04) to the species level, as they showed identical 16S rRNA sequences to other identified strains. The two remaining strains (BD08 and BB02) were identified as *L*. *helveticus* and *B*. *breve* using comparison of their proteomes as described in the Materials and Methods section, thereby confirming the species identification for all 8 strains (S4 Table). In line with previous work by Barrangou *et al*. that showed the lack of genetic polymorphism among *B*. *animalis* subsp. *lactis* strains [60], the genomes of *B*. *animalis* subsp. *lactis* strains BL03 and BI04 appeared to differ only by few SNPs and InDels to each other, indicating that both strains share a recent and clonal ancestor.

**Fig 1:**
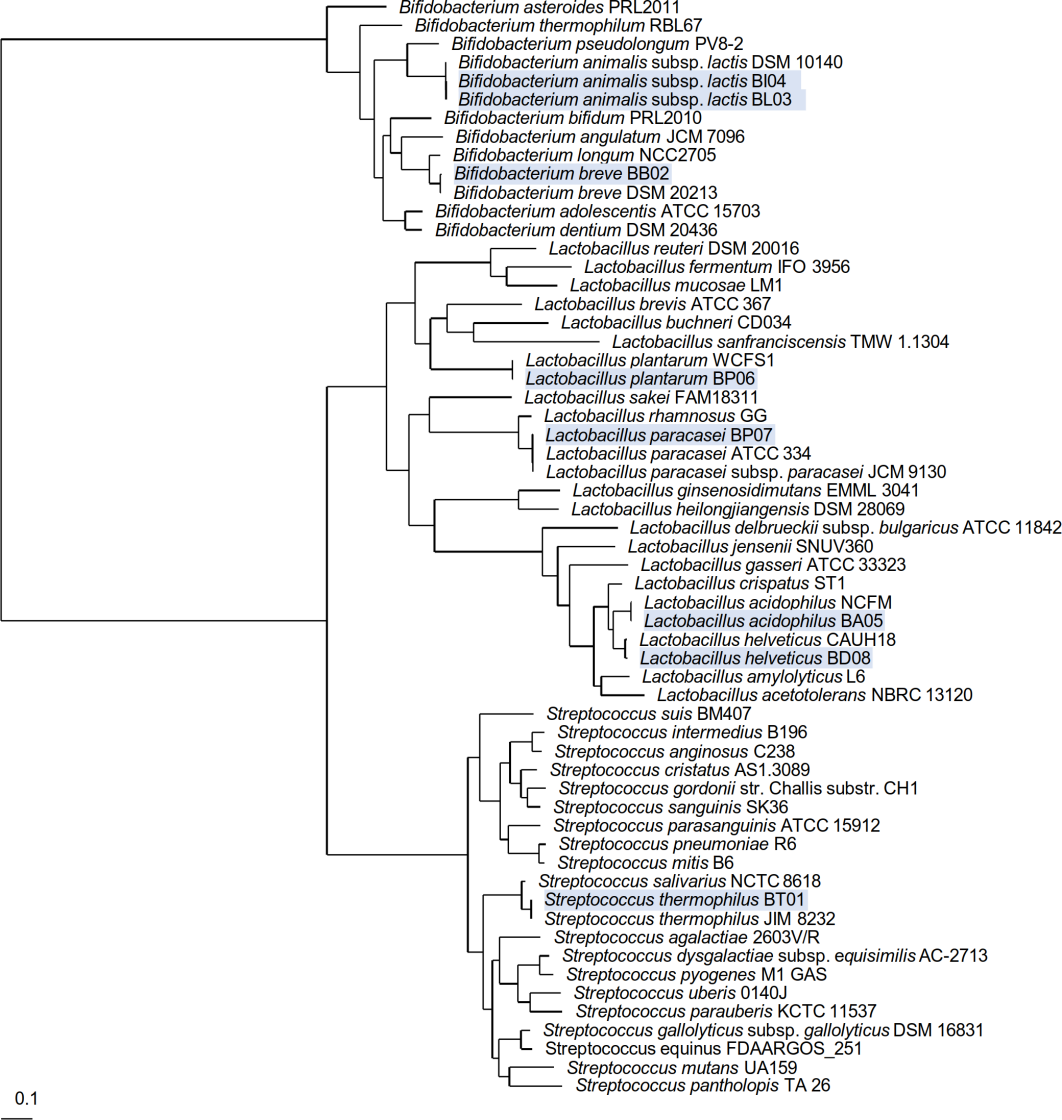
Phylogenetic position of the strains from the VSL#3 product. This phylogenetic tree was generated based on the genome-predicted core proteome shared between the strains from the VSL#3 product and that of a set of relevant reference and representative strains. The position of the eight strains used for the formulation of the VSL#3 product are shaded in blue.

Subsequently, the predicted core proteomes of the 8 VSL#3 strains were compared to a set of relevant reference and clearly positioned in the phylogenetic tree next to the genome of the same species (Fig 1). This final genome-based identification of the 8 VSL#3 strains confirmed the composition of the multispecies product VSL#3 as initially specified by the manufacturer (see S1 and S3 Tables). Further analysis of the genomes and their annotation provided insight in the genome size, GC content, predicted number of genes, including that for rRNAs and tRNAs (Table 1). It is noteworthy that the genome sequence of *S*. *thermophilus* had fewer tRNAs genes compared to other draft genomes sequences. This is, however, possibly due to the genome assembly based on gene context and comparison with the strain *S*. *thermophilus* JIM8232 [61].

**Table 1:** General predicted genomic features of bacterial strains from VSL#3 product.

Bacterial strain	Genome size (Mbp)	Number of genes	Number of tRNA	Number of rRNA	Predicted plasmids
*Lactobacillus helveticus* BD08	1.82	1932	60	3	1
*Lactobacillus paracasei* BP07	2.87	2718	50	2	0
*Lactobacillus plantarum* BP06	3.21	2995	62	3	0
*Lactobacillus acidophilus* BA05	1.97	1852	59	3	0
*Streptococcus thermophilus* BT01	1.81	1894	37	3	2
*Bifidobacterium breve* BB02	2.32	1972	59	3	0
*Bifidobacterium animalis* subsp. *lactis* BL03	1.92	1552	54	3	0
*Bifidobacterium animalis* subsp. *lactis* BI04	1.92	1554	54	3	0

### Mobilome of the multispecies probiotic-marketed product VSL#3

Bacteria are known to harbor various mobile elements within their genomes, such as insertion sequence elements, transposases, plasmids or prophages [62], which impact on the genome plasticity and stability, as previously shown in some LAB [22]. Mobilome analysis may offer insights into the ecological niche and its inhabitants, from which a particular strain has been isolated, i.e. exposure to bacteriophage and other foreign mobile DNA elements. An accurate determination of the mobilome is not possible, since we annotated draft genomes that are in several contigs (see S3 Table) and therefore the number of transposases and insertion sequence (IS) elements may be under/overestimated, as a result of the assembly process. However, our current data provides a preliminary overview on the actual mobilome of each strain analyzed in the present study. Whereas most *Bifidobacterium* strains and *Lactobacillus* strains of the VSL#3 product were predicted to have a relative low number of transposons, *Streptococcus thermophilus* strain BT01 and *Lactobacillus helveticus* strain BD08, in contrast, were estimated to harbor the most, respectively 27 and 24 IS transposons (S1 Fig). IS elements are known to be involved in chromosomal deletions and/or rearrangements, thus playing a role in ecological adaptation and species diversification [22,63–65]. Prophage-associated genes were also observed in some of the strains, including *Lactobacillus paracasei* BP07, *Lactobacillus helveticus* BBP06 and *Bifidobacterium breve* BB02. It has been hypothesized that prophages may be involved in lateral gene transfer, by embarking extra-chromosomal elements into their genome [66].

Detailed analysis of the genomic data also suggested the presence of plasmids in *Streptococcus thermophilus* strain BT01 and *Lactobacillus helveticus* strain BD08, consisting of contigs with coverage higher than 1,000-fold. Specifically, a 33-kb contig found in *Lactobacillus helveticus* strain BD08 is predicted to be of plasmid origin and harbors 24 genes, including genes associated with lactose transport (*lacF*), peptidase activity and also carbohydrate, metal and amino acid transport (a detailed listing of predicted genes present in the plasmid contig from *L*. *helveticus* BD08 is shown in S5 Table). In *Streptococcus thermophilus* strain BT01, two contigs with a respective size of 3.3 and 4.5 kb were also predicted to be of plasmid origin and were virtually identical to two plasmids present in *S*. *thermophilus* strain LMD-9 [67]. Among the genes present the two *S*. *thermophilus* plasmids, some were associated with plasmid replication (NicK), glycosyl-transferases and stress response (acid stress) (S6 Table).

### Defense mechanisms against foreign mobile DNA elements: CRISPR-Cas and R/M systems

**Fig 2:**
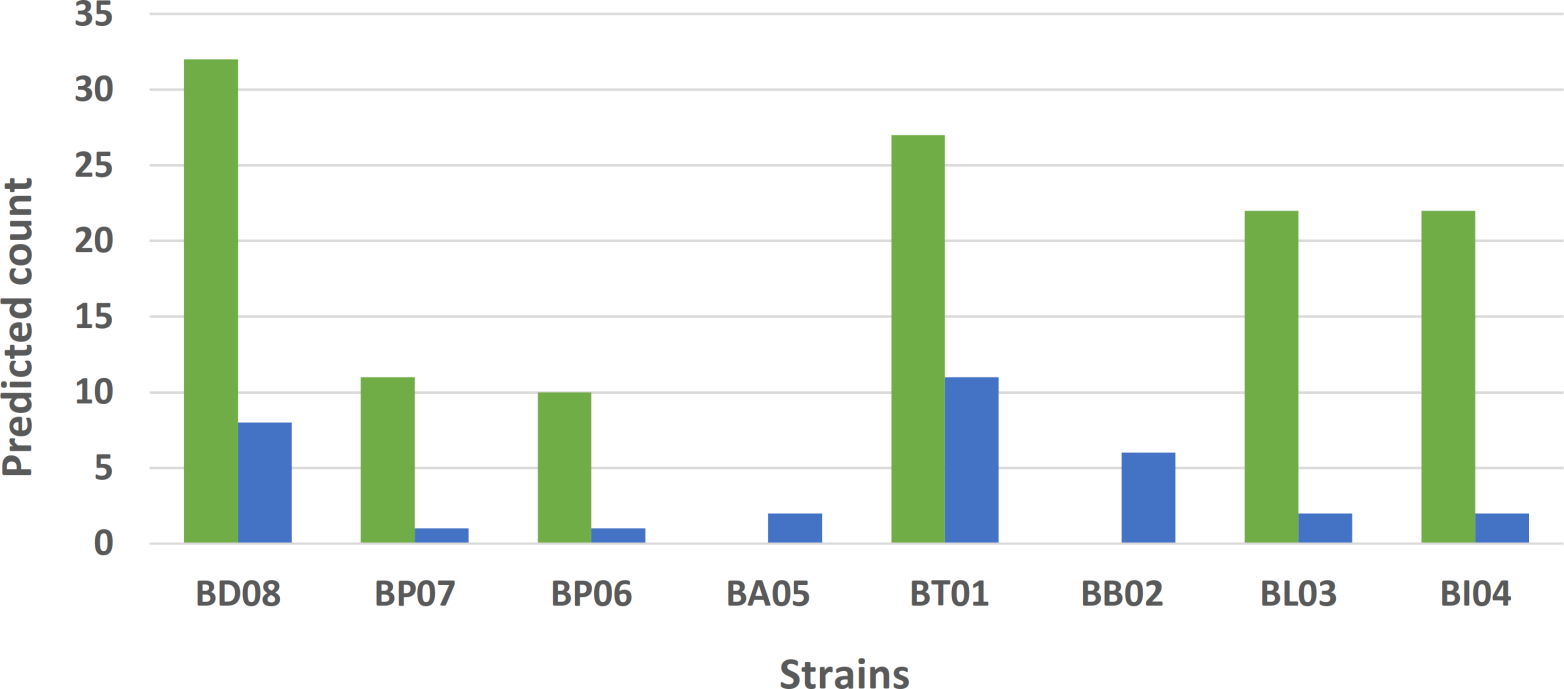
Defense systems of the multi-species probiotic product VSL#3. Number of predicted restriction/modification enzymes (blue) and CRISPR-Cas spacers (green). Strains devoid of CRISPR-Cas loci have a number of spacers equal to zero.

**Fig 3:**
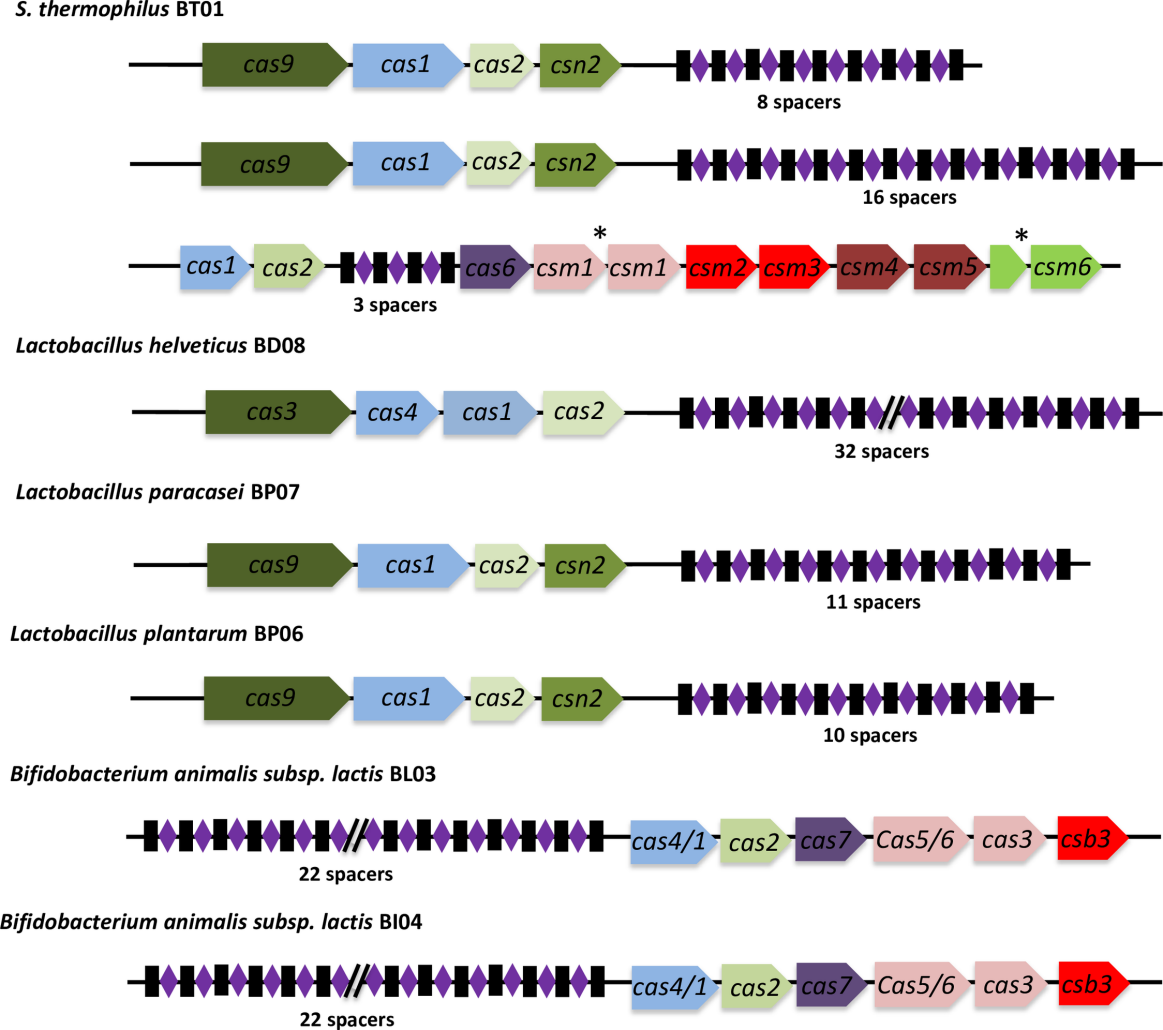
CRISPR-Cas loci identified in the strains of the VSL#3 product. The CRISPR-Cas loci were identified using CRISPRFinder [56] and their gene order and predicted annotations are depicted along with their juxtaposing array of spacers. Legend: *, split gene.

Streptococci, lactobacilli and bifidobacteria are known to be prone to phage infections notably during industrial dairy and other food fermentations as well as in the gut environment [68–70]. Genome annotation revealed that 6 out of the 8 strains harbored at least one CRISPR-Cas locus (Fig 2). Interestingly, *Lactobacillus acidophilus* BP05 and *Bifidobacterium breve* BB02 were devoid of CRISPR-Cas locus. Remarkably, *Streptococcus thermophilus* BT01 harbored three distinct CRISPR-Cas loci (Fig 3). All eight strains also had genes encoding for restriction modification (R/M) systems, *i*.*e*. restriction endonucleases and methyltransferases (Fig 2).

There are a variety of defense mechanisms that protect bacteria against foreign mobile DNA elements, such as plasmids or bacteriophage DNAs. *S*. *thermophilus* BT01 and *L*. *helveticus* BD08 appeared to have acquired diverse and multiple mechanisms to protect themselves from their environment and were predicted to contain CRISPR-Cas loci and R/M systems. This indicates that they may originate from complex ecological habitats co-existing within diverse bacterial and phage communities. Detailed comparative sequence analysis of the CRISPR-Cas spacers revealed homologies with sequences from various known phages or plasmids (S7 Table). In some cases, *i*.*e*. *L*. *helveticus* BD08, only few spacers showed homology to known sequences, suggesting that the potential target of these spacers is yet to be characterized. We also studied the whole repertoire of spacers for all 8 strains and compared this with that of other sequenced bacterial strains. Thus, the three repertoires of *S*. *thermophilus* BT01 spacers were similar to the ones found in the well-studied yoghurt starter strain *S*. *thermophilus* LMD-9 (also called ATCC BAA-491), which is naturally competent [67]. This supports the phylogenetic relatedness and shared origin of these two strains and indicates that *S*. *thermophilus* BT01 may be naturally competent. The spacers found in *L*. *paracasei* BP07 CRISPR-Cas locus were similar to some from several well-studied strains, including the probiotic strain *L*. *casei* BD-II [71] and *L*. *casei* strain W56 [72] that belong to clade A of the *L*. *casei* group [73]. Similar observations were made with *L*. *plantarum* BP06 and *L*. *plantarum* CLP-0611 isolated from kimchi [74] as well as *Bifidobacterium animalis* subsp. *lactis* BI03 or BL04 with *B*. *animalis* subsp. *lactis* strain BF052, a fecal isolate from healthy infants [75]. Blast analysis of the 32 spacer regions of *L*. *helveticus* BD08 gave significant hits with sequenced *L*. *helveticus*, *L*. *amylovorus* and *L*. *gallinarum* strains (10) and, some known *L*. *helveticus* plasmids and phages (3) but the majority (19) could not be matched against the tested phage or plasmid databases, suggesting that the strain derives from a poorly characterized habitat but has encountered some shared environmental DNAs with other lactobacilli.

While some of the strains harbor only few genes encoding the R/M system, others such as *S*. *thermophilus* BT01 and *L*. *helveticus* BD08 were predicted to harbor up to 11 and 8 R/M systems in their respective genome. Interestingly, these two particular strains had CRISPR-Cas loci with a relatively wide repertoire of spacers (32 and 27, respectively). The co-existence of R/M and CRISPR-Cas systems was shown to provide an increased resistance to phages (additive protection) [76] and also to prevent plasmid transfer, as reported in *Enterococcus faecalis* [77].

### Resistome: Putative antibiotic resistance genes and virulence factors

Using the resfams database [53], genome sequences were examined for the presence of genes associated with antibiotic resistance. In all strains, potential antibiotic resistance genes were identified but these were mostly transport systems, potentially functioning as antibiotic efflux pumps, as illustrated in the genome annotation (S3 Table). Five potentially transferable genes associated with a resistance to aminoglycosides, β-lactams and tetracyclin were specifically examined in the genomes of the *Bifidobacterium spp*. and *L*. *acidophilus*. The *tetW* gene identified in the *Bifidobacterium* genomes (BB02, BL03 and BI04) and a potential aminoglycoside aminotransferase gene in strain BI04 were located in the vicinity of a putative transposon gene cassette. The presence of *tetW* gene, associated with a putative transposon, is generally found in other bifidobacteria, such as the widely-consumed *Bifidobacterium animalis* subsp. *lactis* BB-12 [14] and other isolates, but has so far not found to be transferable [78]. Two genes (APH3 and AAC3) associated with the breakdown of aminoglycosides were also identified in the genomes of *L*. *acidophilus* BA05 and *B*. *animalis* subsp. *lactis* BL04 and BI04. The AAC3 of *L*. *acidophilus* BA05 was annotated as being of prophage origin and was also present with complete identity in the genome of the widely consumed strain *L*. *acidophilus* NCFM [7]. Similarly, aminoglycoside phosphotransferases present in *B*. *animalis* subsp. *lactis* BL04 and BI04 could be also found in *Bifidobacterium animalis* subsp. *lactis* BB-12 [14]. The gene coding for class-A β-lactamase in *L*. *plantarum* BP06 similar to the one in *L*. *plantarum* WCFS1 (99% homology) [10]. The class-B β-lactamase gene present in *L*. *plantarum* BP06 was also found in many other *L*. *plantarum* genomes based on blast searches, but not in *L*. *plantarum* WCFS1.

To better address the safety of VSL#3 strains, the antibiotic sensitivity was performed according to the recommendations of the European Food Safety Agency (EFSA) using a micro-dilution method [40]. The data obtained, reported in Table 2, show that all the strains exhibits antibiotic sensitivities within the recommended cut-off values. In only few cases, for kanamycin and chloramphenicol, the MICs measured were higher than the cut-off values for *L*. *paracasei* BP07, *L*. *acidophilus* BA05 and *L*. *helveticus* BD08 used by EFSA [40]. However, while the latter MICs were formally above the recommended cut-off, these fall within the interlaboratory variation of MICs that have been reported for non-enterococcal lactic acid bacteria [41], as illustrated in S2 Table. Hence, we conclude that all the VSL#3 strains respected the safety recommendations of EFSA [40].

**Table 2:** Evaluation of minimal inhibitory concentration (MIC) of antibiotics against VSL#3 strains. The values in bold represent those MICs showing higher values compared to EFSA cut-off (in parenthesis) [40]. n.d. not determined as not required by EFSA since these species show a high level of natural resistance [40].

Bacterial strain	Antibiotic MIC (μg/ml)								
	Ampicillin	Vancomycin	Gentamycin	Kanamycin	Streptomycin	Erythromycin	Clindamycin	Tetracycline	Chloramphenicol
*Lactobacillus helveticus* BD08	0.25	0.5	4	**32**	8	0.25	0.25	1	2
	(1)	(2)	(16)	(16)	(16)	(1)	(1)	(4)	(4)
*Lactobacillus paracasei* BP07	1	n.d.	1	**128**	32	0.25	0.25	4	**8**
	(4)		(32)	(64)	(64)	(1)	(1)	(4)	(4)
*Lactobacillus plantarum* BP06	0.25	n.d.	4	64	n.d.	0.5	0.5	16	1
	(2)		(16)	(64)		(1)	(2)	(32)	(8)
*Lactobacillus acidophilus* BA05	1	1	8	**128**	16	0.25	0.5	4	**8**
	(1)	(2)	(16)	(64)	(16)	(1)	(1)	(4)	(4)
*Streptococcus thermophilus* BT01	0.25	0.5	2	64	32	0.25	0.25	0.5	4
	(2)	(4)	(32)	(64)	(64)	(2)	(2)	(4)	(4)
*Bifidobacterium breve* BB02	1	1	8	n.d.	8	0.25	0.25	2	2
	(2)	(2)	(64)		(128)	(1)	(1)	(8)	(4)
*Bifidobacterium animalis* subsp. *lactis* BL03	0.25	1	32	n.d.	64	0.25	0.25	8	2
	(2)	(2)	(64)		(128)	(1)	(1)	(8)	(4)
*Bifidobacterium animalis* subsp. *lactis* BI04	0.25	1	32	n.d.	128	0.25	0.25	8	2
	(2)	(2)	(64)		(128)	(1)	(1)	(8)	(4)

Summarizing, the present comparative analysis revealed that some potential antibiotic-associated genes found in the strains of the multispecies probiotic product VSL#3 were also present in widely-marketed probiotic strains or well-characterized LAB and bifidobacteria. However, the possible transfer of these potential antibiotic resistance genes to other species remains to be demonstrated.

Our initial analysis also predicted the presence of putative virulence genes in the different genomes by comparative analysis with the VFDB. However, the VFDB is rather broad database and also includes genes that may have an indirect role in virulence. Further inspection of these virulence gene candidates showed that they were related to stress response (*clpC*, *clpE*) or cell wall/CPS biosynthesis (*cps* genes, *galE*). In addition, only single genes were found in specific strains, indicating that the complete pathway required for biosynthesis was absent or at least different from the ones found in pathogenic strains.

### Host adhesion and interaction

**Fig 4:**
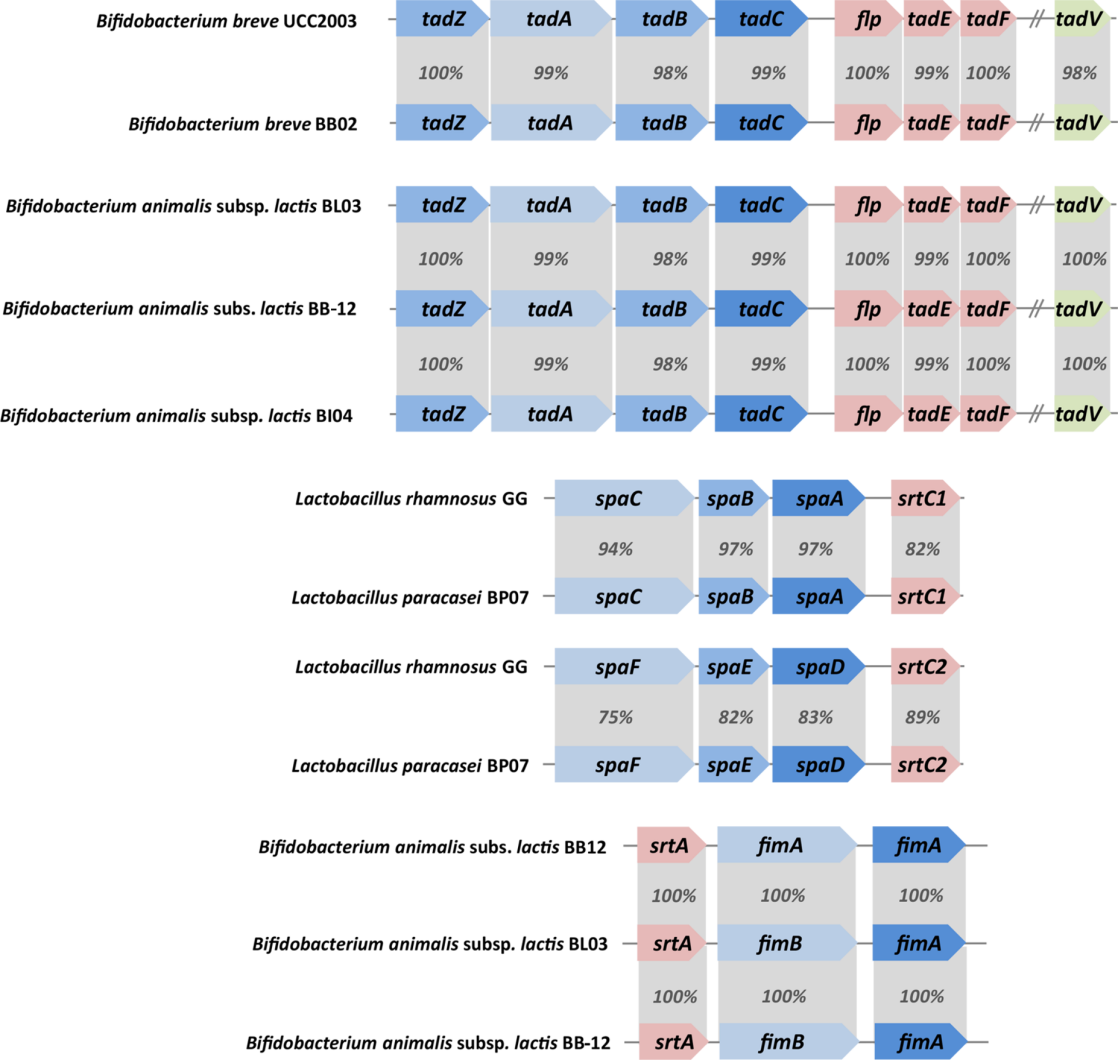
Pilus gene clusters identified in the multispecies product VSL#3. Both Tad pilus and sortase-dependent pilus gene clusters are shown along with a corresponding pilus gene cluster previously characterized [17]. The percentage indicates the degree of amino acid sequence conservation between the different genes.

Gut-adapted bacteria have established various strategies to colonize, interact and signal the host [2,17,79–81]. Genes coding for these surface components were found in the genomes of the strains present in the VSL#3 multispecies product (S3 Table). In addition, some bacteria are decorated with long extra-cellular structures, called pili or fimbriae that have the properties to adhere to the intestinal epithelium [2,17]. In some cases, pili have also been associated with protein secretion and conjugation [82]. Two main types of pili can be found in LAB and bifidobacteria: the Tad pili (also called tight adherence pili, type IVb) and the sortase-dependent pili. In pathogenic bacteria, the tight adherence pili are pivotal in adherence, colonization and also pathogenesis [83]. In *B*. *breve* BB02, *B*. *animalis* subsp. *lactis* BL03 and BI04, gene clusters coding the Tad pili were found (Fig 4). The Tad pilus gene cluster of *B*. *breve* BB02 is highly conserved with the Tad pili of *B*. *breve* UCC2003 that was reported to be involved with gut colonization in mice [17].

Cell-surface associated proteins harboring LPXTG motifs are sortase substrates and many, often large ones, with predicted sizes of up to 3,515 amino acid residues, were found to be encoded by all strains with *L*. *paracasei* BP07, *L*. *plantarum* BP06,and *L*. *acidophilus* BA05 having the highest number of LPXTG proteins (20, 16 and 11, respectively). All predicted LPXTG proteins were compared in details with those of other LAB or bifidobacteria since many have been studied in details for their host interaction capabilities (S8 Table). A number of genes encoding fibronectin binding domain proteins, collagen adhesins, outer membrane proteins, fimbriae or pili were identified in *L*. *helveticus* BD08, *L*. *plantarum* BP06, *L*. *acidophilus* BA05 and *B*. *animalis* subsp. *lactis* BL03 and BI04 with potential roles in host adherence among others [84]. The LPXTG-proteins of *L*. *helveticus* BD08 and *S*. *thermophilus* BT01 were found in all related strains of these species. Similarly, most predicted LPXTG proteins of *L*. *paracasei* BP07 were also found to be encoded by the genome of the dairy strain *L*. *casei* LC2W which belongs to the clade A of the *L*. *casei* group [73]. Its genome shares the highest identity with that of *L*. *paracasei* BP07 and was used as genomic template [85] (see S3 Table). Apart from a small putative collagen adhesion (BP071_02691), two other predicted LPXTG proteins are not encoded by the *L*. *casei* LC2W genome, including the 1269-residue BP071_02238 that is annotated as an adhesin found in many other LAB, and the 1216-residue BP071_01279, which is annotated as a levanase or β-fructosidase and part of the sucrose phosphotransferase system (PTS) gene cluster. Recently, we have shown that homologous genes in an Asian *L*. *plantarum* strain are involved in inuline degradation, suggesting that *L*. *paracasei* BP07 may also degrade this fructose polymer [86].

Along the same lines, all sortase-substrates of *L*. *plantarum* BP06 were also present in the well-studied *L*. *plantarum* WCFS1 and included the large mucus-binding protein with locus tag Lp_1643 that is O-glycosylated by N-acetyl- hexosamine [87]. Its gene contains the KxYKxGKxW peptide in its signal peptide as also two other LPXTG containing genes of *L*. *plantarum* BP06, indicating that these are all secreted via the SecA2-SecY2 system in a glycosylated form and hence may have host-signalling functions [81]. A sortase-dependent pilus production system was found in both *Bifidobacterium animalis* subsp. *lactis* BL03 and BI04, consisting of two genes encoding pilin subunits (termed FimB and FimP) and one sortase gene. However, in *B*. *breve* BB02 these pili genes were also detected but no sortase gene was found. A very recent study showed these sortase-dependent pili to be ubiquitous in *Bifidobacterium* spp. and some coded for FimB subunits that were able to bind starch, xylan and pectin, indicative of a luminal location, while others showed interaction with the host [18,88].

One sortase-dependent pilus gene cluster (PGC) was also found in *L*. *paracasei* BP07 and is highly conserved with the well-characterized *spaCBA-srtC1* pilus gene cluster from *L*. *rhamnosus* GG [2] that has the ability to bind intestinal mucus [2], form biofilms *in vitro* and signal the host [6,89]. However, the region upstream the *spaCBA-srtC1* PGC is not flanked with an insertion element as observed in *L*. *rhamnosus* GG. In the latter, the IS element contains a constitutive promoter. Therefore, the expression of *spaCBA-srtC1* in *L*. *paracasei* BP07, if any, may be controlled and induced by some unknown signaling. A second pilus gene cluster was present in *L*. *paracasei* BP07 with an identical gene order, *i*.*e*. three pilin genes and one sortase gene. Interestingly, the second PGC was highly related to the *spaFED-srtC2* PGC present in *L*. *rhamnosus* GG (Fig 4).

Genes coding for S-layer proteins were also found in the genomes of *L*. *helveticus* BD08 and *L*. *paracasei* BP07 and possibly play a similar role as in other LAB, *i*.*e*. signaling dendritic cells and T-cell functions, bacterial adherence or enzymatic functions [8,90]. In *L*. *acidophilus* BA05 and *L*. *helveticus* BD08 for example, S-layer associated proteins PrtX and SlpA respectively may be involved in bacteria-host interactions and stimulation of the immune response, as previously described in *L*. *acidophilus* NCFM and *L*. *helveticus* MIMLh5 [91,92]. A complete urease operon was identified in *S*. *thermophilus* BT01 genome showing high identity with *ure* operons identified in *S*. *thermophilus* strains and available in the GenBank database. Although urease is associated with the pathogenesis of several bacteria, the human gut microbiota urease is considered a health-related factor [93] by modulating the nitrogen availability of gut microbiota and host [94]. Moreover, urease activity is present in other well characterized probiotic strains such as *Lactobacillus reuteri* [95] and *S*. *salivarius* [96]. An active urease should be therefore considered a peculiar probiotic trait of the multispecies product VSL#3.

## Concluding remarks

In recent years, the globally-marketed multispecies product VSL#3 has been subject to numerous clinical trials and studies that demonstrated its health beneficial properties for the human host. The lack of genomic information relating to the strains composing the VSL#3 product, however, limited a further understanding of its mode of action and efficacy in the human gut. In the present work, we genomically characterized the 8 different strains present in VSL#3. Our results confirmed and extended the species designation as specified previously and provided a rational basis for future studies of probiotic functions, safety, and ecological fitness. The genome sequences will be instrumental in understanding the mechanisms by which the different strains may interact with each other, other intestinal bacteria and the human host. Several candidate genes involved in these processes have been identified and discussed here, such as Tad pili, sortase-dependent pili, mucus binding proteins, some even glycosylated, and S-layer proteins that were previously studied in related organisms. It is of interest that some of the strains analyzed in this work showed high genomic similarities with well-characterized industrial strains or model strains where particular probiotic traits or modes of action towards the host have been investigated in detail. In most cases, these studies were conducted on single species, and hence the original and unique combination of *Lactobacillus*, *Streptococcus* and *Bifidobacterium* spp. in the VSL#3 product suggests possible complementary and synergistic effects in the gut that will need to be further investigated using both functional genomic approaches and experimental studies.

## Supporting information

S1 Fig Predicted number of transposons in each sequenced genome. (TIF)

S1 Table List of bacterial strains present in the multispecies probiotic-marketed product VSL#3 and growth conditions used in the present study. (DOCX)

S2 Table Sensitivity of *Lactobacillus paracasei* LMG12586 to various antibiotics compared to that reported by ISO10932/IDF223 2010. (DOCX)

S3 Table Genome sequencing statistics. (XLSX)

S4 Table Genome annotations of the different strains sequenced in this study. (XLSX)

S5 Table Genes present in *L*. *helveticus* BD08 plasmid. (XLSX)

S6 Table Genes present in *S*. *thermophilus* BT01 plasmids. (XLSX)

S7 Table BLAST analysis of the spacer sequences identified in the different CRISPR-Cas loci. (XLSX)

S8 Table List of predicted LPXTG proteins identified in the present study. (XLSX)

## References

[1] Hill C, Guarner F, Reid G, Gibson GR, Merenstein DJ, et al (2014) Expert consensus document. The International Scientific Association for Probiotics and Prebiotics consensus statement on the scope and appropriate use of the term probiotic. Nat Rev Gastroenterol Hepatol 11: 506–514. doi: 10.1038/nrgastro.2014.66 24912386 10.1038/nrgastro.2014.66

[2] Kankainen M, Paulin L, Tynkkynen S, von Ossowski I, Reunanen J, et al (2009) Comparative genomic analysis of *Lactobacillus rhamnosus* GG reveals pili containing a human- mucus binding protein. Proc Natl Acad Sci U S A 106: 17193–17198. doi: 10.1073/pnas.0908876106 19805152 10.1073/pnas.0908876106 PMC2746127

[3] Douillard FP, Ribbera A, Kant R, Pietilä TE, Järvinen HM, et al (2013) Comparative genomic and functional analysis of 100 *Lactobacillus rhamnosus* strains and their comparison with strain GG. PLoS Genet 9: e1003683 doi: 10.1371/journal.pgen.1003683 23966868 10.1371/journal.pgen.1003683 PMC3744422

[4] Nissilä E, Douillard FP, Ritari J, Paulin L, Järvinen HM, et al (2017) Genotypic and phenotypic diversity of *Lactobacillus rhamnosus* clinical isolates, their comparison with strain GG and their recognition by complement system. PLoS One 12: e0176739 doi: 10.1371/journal.pone.0176739 28493885 10.1371/journal.pone.0176739 PMC5426626

[5] Tytgat HL, Douillard FP, Reunanen J, Rasinkangas P, Hendrickx AP, et al (2016) *Lactobacillus rhamnosus* GG outcompetes *Enterococcus faecium* via mucus-binding pili: Evidence for a novel and heterospecific probiotic mechanism. Appl Environ Microbiol 82: 5756–5762. doi: 10.1128/AEM.01243-16 27422834 10.1128/AEM.01243-16 PMC5038030

[6] Tytgat HL, van Teijlingen NH, Sullan RM, Douillard FP, Rasinkangas P, et al (2016) Probiotic gut microbiota isolate interacts with dendritic cells via glycosylated heterotrimeric pili. PLoS One 11: e0151824 doi: 10.1371/journal.pone.0151824 26985831 10.1371/journal.pone.0151824 PMC4795749

[7] Altermann E, Russell WM, Azcarate-Peril MA, Barrangou R, Buck BL, et al (2005) Complete genome sequence of the probiotic lactic acid bacterium *Lactobacillus acidophilus* NCFM. Proc Natl Acad Sci U S A 102: 3906–3912. doi: 10.1073/pnas.0409188102 15671160 10.1073/pnas.0409188102 PMC554803

[8] Konstantinov SR, Smidt H, de Vos WM, Bruijns SC, Singh SK, et al (2008) S layer protein A of *Lactobacillus acidophilus* NCFM regulates immature dendritic cell and T cell functions. Proc Natl Acad Sci U S A 105: 19474–19479. doi: 10.1073/pnas.0810305105 19047644 10.1073/pnas.0810305105 PMC2592362

[9] Johnson B, Selle K, O'Flaherty S, Goh YJ, Klaenhammer T (2013) Identification of extracellular surface-layer associated proteins in *Lactobacillus acidophilus* NCFM. Microbiology 159: 2269–2282. doi: 10.1099/mic.0.070755-0 24002751 10.1099/mic.0.070755-0 PMC3836491

[10] Kleerebezem M, Boekhorst J, van Kranenburg R, Molenaar D, Kuipers OP, et al (2003) Complete genome sequence of *Lactobacillus plantarum* WCFS1. Proc Natl Acad Sci U S A 100: 1990–1995. doi: 10.1073/pnas.0337704100 12566566 10.1073/pnas.0337704100 PMC149946

[11] van den Nieuwboer M, van Hemert S, Claassen E, de Vos WM (2016) *Lactobacillus plantarum* WCFS1 and its host interaction: a dozen years after the genome. Microb Biotechnol 9: 452–465. doi: 10.1111/1751-7915.12368 27231133 10.1111/1751-7915.12368 PMC4919987

[12] van Baarlen P, Troost FJ, van Hemert S, van der Meer C, de Vos WM, et al (2009) Differential NF-kappaB pathways induction by *Lactobacillus plantarum* in the duodenum of healthy humans correlating with immune tolerance. Proc Natl Acad Sci U S A 106: 2371–2376. doi: 10.1073/pnas.0809919106 19190178 10.1073/pnas.0809919106 PMC2650163

[13] Mujagic Z, de Vos P, Boekschoten MV, Govers C, Pieters HH, et al (2017) The effects of *Lactobacillus plantarum* on small intestinal barrier function and mucosal gene transcription; a randomized double-blind placebo controlled trial. Sci Rep 7: 40128 doi: 10.1038/srep40128 28045137 10.1038/srep40128 PMC5206730

[14] Garrigues C, Johansen E, Pedersen MB (2010) Complete genome sequence of *Bifidobacterium animalis* subsp. *lactis* BB-12, a widely consumed probiotic strain. J Bacteriol 192: 2467–2468. doi: 10.1128/JB.00109-10 20190051 10.1128/JB.00109-10 PMC2863482

[15] Loquasto JR, Barrangou R, Dudley EG, Stahl B, Chen C, et al (2013) *Bifidobacterium animalis* subsp. *lactis* ATCC 27673 is a genomically unique strain within its conserved subspecies. Appl Environ Microbiol 79: 6903–6910. doi: 10.1128/AEM.01777-13 23995933 10.1128/AEM.01777-13 PMC3811525

[16] Ventura M, O'Flaherty S, Claesson MJ, Turroni F, Klaenhammer TR, et al (2009) Genome-scale analyses of health-promoting bacteria: probiogenomics. Nat Rev Microbiol 7: 61–71. doi: 10.1038/nrmicro2047 19029955 10.1038/nrmicro2047

[17] O'Connell Motherway M, Zomer A, Leahy SC, Reunanen J, Bottacini F, et al (2011) Functional genome analysis of *Bifidobacterium breve* UCC2003 reveals type IVb tight adherence (Tad) pili as an essential and conserved host-colonization factor. Proc Natl Acad Sci U S A 108: 11217–11222. doi: 10.1073/pnas.1105380108 21690406 10.1073/pnas.1105380108 PMC3131351

[18] Milani C, Mangifesta M, Mancabelli L, Lugli GA, Mancino W, et al (2017) The sortase-dependent fimbriome of the genus *Bifidobacterium*: extracellular structures with potential to modulate microbe-host dialogue. Appl Environ Microbiol. 83(19):e01295–17. doi: 10.1128/AEM.01295-17 28754709 10.1128/AEM.01295-17 PMC5601332

[19] Fanning S, Hall LJ, van Sinderen D (2012) *Bifidobacterium breve* UCC2003 surface exopolysaccharide production is a beneficial trait mediating commensal-host interaction through immune modulation and pathogen protection. Gut Microbes 3: 420–425. doi: 10.4161/gmic.20630 22713271 10.4161/gmic.20630

[20] Douillard FP, de Vos WM (2014) Functional genomics of lactic acid bacteria: from food to health. Microb Cell Fact 13 Suppl 1: S8. 25186768 10.1186/1475-2859-13-S1-S8 PMC4155825

[21] Sun Z, Harris HM, McCann A, Guo C, Argimon S, et al (2015) Expanding the biotechnology potential of lactobacilli through comparative genomics of 213 strains and associated genera. Nat Commun 6: 8322 doi: 10.1038/ncomms9322 26415554 10.1038/ncomms9322 PMC4667430

[22] Douillard FP, Ribbera A, Xiao K, Ritari J, Rasinkangas P, et al (2016) Polymorphisms, chromosomal rearrangements, and mutator phenotype development during experimental evolution of *Lactobacillus rhamnosus* GG. Appl Environ Microbiol 82: 3783–3792. doi: 10.1128/AEM.00255-16 27084020 10.1128/AEM.00255-16 PMC4907198

[23] Douillard FP, Kant R, Ritari J, Paulin L, Palva A, et al (2013) Comparative genome analysis of *Lactobacillus casei* strains isolated from Actimel and Yakult products reveals marked similarities and points to a common origin. Microb Biotechnol 6: 576–587. doi: 10.1111/1751-7915.12062 23815335 10.1111/1751-7915.12062 PMC3918159

[24] Mimura T, Rizzello F, Helwig U, Poggioli G, Schreiber S, et al (2004) Once daily high dose probiotic therapy (VSL#3) for maintaining remission in recurrent or refractory pouchitis. Gut 53: 108–114. 14684584 10.1136/gut.53.1.108 PMC1773918

[25] Sood A, Midha V, Makharia GK, Ahuja V, Singal D, et al (2009) The probiotic preparation, VSL#3 induces remission in patients with mild-to-moderately active ulcerative colitis. Clin Gastroenterol Hepatol 7: 1202–1209, 1209 e1201. doi: 10.1016/j.cgh.2009.07.016 19631292 10.1016/j.cgh.2009.07.016

[26] Kim HJ, Vazquez Roque MI, Camilleri M, Stephens D, Burton DD, et al (2005) A randomized controlled trial of a probiotic combination VSL# 3 and placebo in irritable bowel syndrome with bloating. Neurogastroenterol Motil 17: 687–696. doi: 10.1111/j.1365-2982.2005.00695.x 16185307 10.1111/j.1365-2982.2005.00695.x

[27] Caballero-Franco C, Keller K, De Simone C, Chadee K (2007) The VSL#3 probiotic formula induces mucin gene expression and secretion in colonic epithelial cells. Am J Physiol Gastrointest Liver Physiol 292: G315–322. doi: 10.1152/ajpgi.00265.2006 16973917 10.1152/ajpgi.00265.2006

[28] Massi M, Vitali B, Federici F, Matteuzzi D, Brigidi P (2004) Identification method based on PCR combined with automated ribotyping for tracking probiotic Lactobacillus strains colonizing the human gut and vagina. J Appl Microbiol 96: 777–786. 15012816 10.1111/j.1365-2672.2004.02228.x

[29] Vitali B, Candela M, Matteuzzi D, Brigidi P (2003) Quantitative detection of probiotic Bifidobacterium strains in bacterial mixtures by using real-time PCR. Syst Appl Microbiol 26: 269–276. doi: 10.1078/072320203322346128 12866854 10.1078/072320203322346128

[30] Lewis ZT, Shani G, Masarweh CF, Popovic M, Frese SA, et al (2016) Validating bifidobacterial species and subspecies identity in commercial probiotic products. Pediatr Res 79: 445–452. doi: 10.1038/pr.2015.244 26571226 10.1038/pr.2015.244 PMC4916961

[31] Vandamme P, Peeters C (2014) Time to revisit polyphasic taxonomy. Antonie Van Leeuwenhoek 106: 57–65. doi: 10.1007/s10482-014-0148-x 24633913 10.1007/s10482-014-0148-x

[32] Tursi A, Brandimarte G, Papa A, Giglio A, Elisei W, et al (2010) Treatment of relapsing mild-to-moderate ulcerative colitis with the probiotic VSL#3 as adjunctive to a standard pharmaceutical treatment: a double-blind, randomized, placebo-controlled study. Am J Gastroenterol 105: 2218–2227. doi: 10.1038/ajg.2010.218 20517305 10.1038/ajg.2010.218 PMC3180711

[33] Alisi A, Bedogni G, Baviera G, Giorgio V, Porro E, et al (2014) Randomised clinical trial: The beneficial effects of VSL#3 in obese children with non-alcoholic steatohepatitis. Aliment Pharmacol Ther 39: 1276–1285. doi: 10.1111/apt.12758 24738701 10.1111/apt.12758 PMC4046270

[34] Dhiman RK, Rana B, Agrawal S, Garg A, Chopra M, et al (2014) Probiotic VSL#3 reduces liver disease severity and hospitalization in patients with cirrhosis: a randomized, controlled trial. Gastroenterology 147: 1327–1337 e1323. doi: 10.1053/j.gastro.2014.08.031 25450083 10.1053/j.gastro.2014.08.031

[35] Rajkumar H, Mahmood N, Kumar M, Varikuti SR, Challa HR, et al (2014) Effect of probiotic (VSL#3) and omega-3 on lipid profile, insulin sensitivity, inflammatory markers, and gut colonization in overweight adults: a randomized, controlled trial. Mediators Inflamm 2014: 348959 doi: 10.1155/2014/348959 24795503 10.1155/2014/348959 PMC3984795

[36] Hart AL, Lammers K, Brigidi P, Vitali B, Rizzello F, et al (2004) Modulation of human dendritic cell phenotype and function by probiotic bacteria. Gut 53: 1602–1609. doi: 10.1136/gut.2003.037325 15479680 10.1136/gut.2003.037325 PMC1774301

[37] Mariman R, Tielen F, Koning F, Nagelkerken L (2014) The probiotic mixture VSL#3 dampens LPS-induced chemokine expression in human dendritic cells by inhibition of STAT-1 phosphorylation. PLoS One 9: e115676 doi: 10.1371/journal.pone.0115676 25546330 10.1371/journal.pone.0115676 PMC4278714

[38] Pagnini C, Saeed R, Bamias G, Arseneau KO, Pizarro TT, et al (2010) Probiotics promote gut health through stimulation of epithelial innate immunity. Proc Natl Acad Sci U S A 107: 454–459. doi: 10.1073/pnas.0910307107 20018654 10.1073/pnas.0910307107 PMC2806692

[39] Kahlmeter G, Brown DF, Goldstein FW, MacGowan AP, Mouton JW, et al (2003) European harmonization of MIC breakpoints for antimicrobial susceptibility testing of bacteria. J Antimicrob Chemother 52: 145–148. doi: 10.1093/jac/dkg312 12837738 10.1093/jac/dkg312

[40] EFSA Panel on Additives Products or Substances used in Animal Feed (FEEDAP) (2012) Guidance on the assessment of bacterial susceptibility to antimicrobials of human and veterinary importance. EFSA Journal 10(6): 2740.

[41] International Dairy Federation (2012) Milk and milk products—Determination of the minimal inhibitory concentration (MIC) of antibiotics applicable to bifidobacteria and non-enterococcal lactic acid bacteria (LAB) Brussels, Belgium: International Dairy Federation.

[42] Walker BJ, Abeel T, Shea T, Priest M, Abouelliel A, et al (2014) Pilon: an integrated tool for comprehensive microbial variant detection and genome assembly improvement. PLoS One 9: e112963 doi: 10.1371/journal.pone.0112963 25409509 10.1371/journal.pone.0112963 PMC4237348

[43] Boetzer M, Henkel CV, Jansen HJ, Butler D, Pirovano W (2011) Scaffolding pre-assembled contigs using SSPACE. Bioinformatics 27: 578–579. doi: 10.1093/bioinformatics/btq683 21149342 10.1093/bioinformatics/btq683

[44] Boetzer M, Pirovano W (2012) Toward almost closed genomes with GapFiller. Genome Biol 13: R56 doi: 10.1186/gb-2012-13-6-r56 22731987 10.1186/gb-2012-13-6-r56 PMC3446322

[45] Seemann T (2014) Prokka: rapid prokaryotic genome annotation. Bioinformatics 30: 2068–2069. doi: 10.1093/bioinformatics/btu153 24642063 10.1093/bioinformatics/btu153

[46] Hyatt D, Chen GL, Locascio PF, Land ML, Larimer FW, et al (2010) Prodigal: prokaryotic gene recognition and translation initiation site identification. BMC Bioinformatics 11: 119 doi: 10.1186/1471-2105-11-119 20211023 10.1186/1471-2105-11-119 PMC2848648

[47] Laslett D, Canback B (2004) ARAGORN, a program to detect tRNA genes and tmRNA genes in nucleotide sequences. Nucleic Acids Res 32: 11–16. doi: 10.1093/nar/gkh152 14704338 10.1093/nar/gkh152 PMC373265

[48] Petersen TN, Brunak S, von Heijne G, Nielsen H (2011) SignalP 4.0: discriminating signal peptides from transmembrane regions. Nat Methods 8: 785–786. doi: 10.1038/nmeth.1701 21959131 10.1038/nmeth.1701

[49] Mistry J, Finn RD, Eddy SR, Bateman A, Punta M (2013) Challenges in homology search: HMMER3 and convergent evolution of coiled-coil regions. Nucleic Acids Res 41: e121 doi: 10.1093/nar/gkt263 23598997 10.1093/nar/gkt263 PMC3695513

[50] DeSantis TZ, Hugenholtz P, Larsen N, Rojas M, Brodie EL, et al (2006) Greengenes, a chimera-checked 16S rRNA gene database and workbench compatible with ARB. Appl Environ Microbiol 72: 5069–5072. doi: 10.1128/AEM.03006-05 16820507 10.1128/AEM.03006-05 PMC1489311

[51] Li L, Stoeckert CJ Jr., Roos DS (2003) OrthoMCL: identification of ortholog groups for eukaryotic genomes. Genome Res 13: 2178–2189. doi: 10.1101/gr.1224503 12952885 10.1101/gr.1224503 PMC403725

[52] Smokvina T, Wels M, Polka J, Chervaux C, Brisse S, et al (2013) *Lactobacillus paracasei* comparative genomics: towards species pan-genome definition and exploitation of diversity. PLoS One 8: e68731 doi: 10.1371/journal.pone.0068731 23894338 10.1371/journal.pone.0068731 PMC3716772

[53] Gibson MK, Forsberg KJ, Dantas G (2015) Improved annotation of antibiotic resistance determinants reveals microbial resistomes cluster by ecology. ISME J 9: 207–216. doi: 10.1038/ismej.2014.106 25003965 10.1038/ismej.2014.106 PMC4274418

[54] Chen L, Yang J, Yu J, Yao Z, Sun L, et al (2005) VFDB: a reference database for bacterial virulence factors. Nucleic Acids Res 33: D325–328. doi: 10.1093/nar/gki008 15608208 10.1093/nar/gki008 PMC539962

[55] Altschul SF, Gish W, Miller W, Myers EW, Lipman DJ (1990) Basic local alignment search tool. J Mol Biol 215: 403–410. 2231712 10.1016/S0022-2836(05)80360-2

[56] Grissa I, Vergnaud G, Pourcel C (2007) CRISPRFinder: a web tool to identify clustered regularly interspaced short palindromic repeats. Nucleic Acids Res 35: W52–57. doi: 10.1093/nar/gkm360 17537822 10.1093/nar/gkm360 PMC1933234

[57] Biswas A, Gagnon JN, Brouns SJ, Fineran PC, Brown CM (2013) CRISPRTarget: bioinformatic prediction and analysis of crRNA targets. RNA Biol 10: 817–827. doi: 10.4161/rna.24046 23492433 10.4161/rna.24046 PMC3737339

[58] Boekhorst J, de Been MW, Kleerebezem M, Siezen RJ (2005) Genome-wide detection and analysis of cell wall-bound proteins with LPxTG-like sorting motifs. J Bacteriol 187: 4928–4934. doi: 10.1128/JB.187.14.4928-4934.2005 15995208 10.1128/JB.187.14.4928-4934.2005 PMC1169495

[59] Litou ZI, Bagos PG, Tsirigos KD, Liakopoulos TD, Hamodrakas SJ (2008) Prediction of cell wall sorting signals in gram-positive bacteria with a hidden markov model: application to complete genomes. J Bioinform Comput Biol 6: 387–401. 18464329 10.1142/s0219720008003382

[60] Barrangou R, Briczinski EP, Traeger LL, Loquasto JR, Richards M, et al (2009) Comparison of the complete genome sequences of *Bifidobacterium animalis* subsp. *lactis* DSM 10140 and Bl-04. J Bacteriol 191: 4144–4151. doi: 10.1128/JB.00155-09 19376856 10.1128/JB.00155-09 PMC2698493

[61] Delorme C, Bartholini C, Luraschi M, Pons N, Loux V, et al (2011) Complete genome sequence of the pigmented *Streptococcus thermophilus* strain JIM8232. J Bacteriol 193: 5581–5582. doi: 10.1128/JB.05404-11 21914889 10.1128/JB.05404-11 PMC3187418

[62] Siefert JL (2009) Defining the mobilome. Methods Mol Biol 532: 13–27. doi: 10.1007/978-1-60327-853-9_2 19271177 10.1007/978-1-60327-853-9_2

[63] Hoffmann FG, McGuire LP, Counterman BA, Ray DA (2015) Transposable elements and small RNAs: Genomic fuel for species diversity. Mob Genet Elements 5: 63–66. doi: 10.1080/2159256X.2015.1066919 26904375 10.1080/2159256X.2015.1066919 PMC4743710

[64] Casacuberta E, Gonzalez J (2013) The impact of transposable elements in environmental adaptation. Mol Ecol 22: 1503–1517. doi: 10.1111/mec.12170 23293987 10.1111/mec.12170

[65] Stapley J, Santure AW, Dennis SR (2015) Transposable elements as agents of rapid adaptation may explain the genetic paradox of invasive species. Mol Ecol 24: 2241–2252. doi: 10.1111/mec.13089 25611725 10.1111/mec.13089

[66] Canchaya C, Fournous G, Chibani-Chennoufi S, Dillmann ML, Brussow H (2003) Phage as agents of lateral gene transfer. Curr Opin Microbiol 6: 417–424. 12941415 10.1016/s1369-5274(03)00086-9

[67] Lecomte X, Gagnaire V, Briard-Bion V, Jardin J, Lortal S, et al (2014) The naturally competent strain *Streptococcus thermophilus* LMD-9 as a new tool to anchor heterologous proteins on the cell surface. Microb Cell Fact 13: 82 doi: 10.1186/1475-2859-13-82 24902482 10.1186/1475-2859-13-82 PMC4076053

[68] Marco MB, Moineau S, Quiberoni A (2012) Bacteriophages and dairy fermentations. Bacteriophage 2: 149–158. doi: 10.4161/bact.21868 23275866 10.4161/bact.21868 PMC3530524

[69] Garneau JE, Moineau S (2011) Bacteriophages of lactic acid bacteria and their impact on milk fermentations. Microb Cell Fact 10 Suppl 1: S20. 21995802 10.1186/1475-2859-10-S1-S20 PMC3231927

[70] Ventura M, Sozzi T, Turroni F, Matteuzzi D, van Sinderen D (2011) The impact of bacteriophages on probiotic bacteria and gut microbiota diversity. Genes Nutr 6: 205–207. doi: 10.1007/s12263-010-0188-4 21484155 10.1007/s12263-010-0188-4 PMC3145054

[71] Ai L, Chen C, Zhou F, Wang L, Zhang H, et al (2011) Complete genome sequence of the probiotic strain *Lactobacillus casei* BD-II. J Bacteriol 193: 3160–3161. doi: 10.1128/JB.00421-11 21478345 10.1128/JB.00421-11 PMC3133187

[72] Hochwind K, Weinmaier T, Schmid M, van Hemert S, Hartmann A, et al (2012) Draft genome sequence of *Lactobacillus casei* W56. J Bacteriol 194: 6638 doi: 10.1128/JB.01386-12 23144392 10.1128/JB.01386-12 PMC3497524

[73] Wuyts S, Wittouck S, De Boeck I, Allonsius CN, Pasolli E, et al (2017) Large-scale phylogenomics of the *Lactobacillus casei* group highlights taxonomic inconsistencies and reveals novel clade-associated features. mSystems 2(4) e00061–17. doi: 10.1128/mSystems.00061-17 28845461 10.1128/mSystems.00061-17 PMC5566788

[74] Jang SE, Han MJ, Kim SY, Kim DH (2014) *Lactobacillus plantarum* CLP-0611 ameliorates colitis in mice by polarizing M1 to M2-like macrophages. Int Immunopharmacol 21: 186–192. doi: 10.1016/j.intimp.2014.04.021 24815859 10.1016/j.intimp.2014.04.021

[75] Charnchai P, Jantama SS, Prasitpuriprecha C, Kanchanatawee S, Jantama K (2016) Effects of the food manufacturing chain on the viability and functionality of *Bifidobacterium animalis* through simulated gastrointestinal conditions. PLoS One 11: e0157958 doi: 10.1371/journal.pone.0157958 27333286 10.1371/journal.pone.0157958 PMC4917081

[76] Dupuis ME, Villion M, Magadan AH, Moineau S (2013) CRISPR-Cas and restriction-modification systems are compatible and increase phage resistance. Nat Commun 4: 2087 doi: 10.1038/ncomms3087 23820428 10.1038/ncomms3087

[77] Price VJ, Huo W, Sharifi A, Palmer KL (2016) CRISPR-Cas and Restriction-Modification act additively against conjugative antibiotic resistance plasmid transfer in *Enterococcus faecalis*. mSphere 1 (3) e00064–16. doi: 10.1128/mSphere.00064-16 27303749 10.1128/mSphere.00064-16 PMC4894674

[78] Gueimonde M, Florez AB, van Hoek AH, Stuer-Lauridsen B, Stroman P, et al (2010) Genetic basis of tetracycline resistance in *Bifidobacterium animalis* subsp. *lactis*. Appl Environ Microbiol 76: 3364–3369. doi: 10.1128/AEM.03096-09 20348299 10.1128/AEM.03096-09 PMC2869156

[79] Perez PF, Minnaard Y, Disalvo EA, De Antoni GL (1998) Surface properties of bifidobacterial strains of human origin. Appl Environ Microbiol 64: 21–26. 9435057 10.1128/aem.64.1.21-26.1998 PMC124666

[80] Hidalgo-Cantabrana C, Sanchez B, Milani C, Ventura M, Margolles A, et al (2014) Genomic overview and biological functions of exopolysaccharide biosynthesis in *Bifidobacterium* spp. Appl Environ Microbiol 80: 9–18. doi: 10.1128/AEM.02977-13 24123746 10.1128/AEM.02977-13 PMC3910989

[81] Tytgat HL, de Vos WM (2016) Sugar Coating the Envelope: Glycoconjugates for microbe-host crosstalk. Trends Microbiol 24: 853–861. doi: 10.1016/j.tim.2016.06.004 27374775 10.1016/j.tim.2016.06.004

[82] Filloux A (2010) A variety of bacterial pili involved in horizontal gene transfer. J Bacteriol 192: 3243–3245. doi: 10.1128/JB.00424-10 20418394 10.1128/JB.00424-10 PMC2897649

[83] Mandlik A, Swierczynski A, Das A, Ton-That H (2008) Pili in Gram-positive bacteria: assembly, involvement in colonization and biofilm development. Trends Microbiol 16: 33–40. doi: 10.1016/j.tim.2007.10.010 18083568 10.1016/j.tim.2007.10.010 PMC2841691

[84] Sengupta R, Altermann E, Anderson RC, McNabb WC, Moughan PJ, et al (2013) The role of cell surface architecture of lactobacilli in host-microbe interactions in the gastrointestinal tract. Mediators Inflamm 2013: 237921 doi: 10.1155/2013/237921 23576850 10.1155/2013/237921 PMC3610365

[85] Chen C, Ai L, Zhou F, Wang L, Zhang H, et al (2011) Complete genome sequence of the probiotic bacterium *Lactobacillus casei* LC2W. J Bacteriol 193: 3419–3420. doi: 10.1128/JB.05017-11 21515769 10.1128/JB.05017-11 PMC3133282

[86] Buntin N, Hongpattarakere T, Ritari J, Douillard FP, Paulin L, et al (2017) An inducible operon is involved in inulin utilization in *Lactobacillus plantarum* strains, as revealed by comparative proteogenomics and metabolic profiling. Appl Environ Microbiol 83(2):e02402–16. doi: 10.1128/AEM.02402-16 27815279 10.1128/AEM.02402-16 PMC5203619

[87] Fredriksen L, Moen A, Adzhubei AA, Mathiesen G, Eijsink VG, et al (2013) *Lactobacillus plantarum* WCFS1 O-linked protein glycosylation: an extended spectrum of target proteins and modification sites detected by mass spectrometry. Glycobiology 23: 1439–1451. doi: 10.1093/glycob/cwt071 24000282 10.1093/glycob/cwt071

[88] Turroni F, Serafini F, Foroni E, Duranti S, O'Connell Motherway M, et al (2013) Role of sortase-dependent pili of *Bifidobacterium bifidum* PRL2010 in modulating bacterium-host interactions. Proc Natl Acad Sci U S A 110: 11151–11156. doi: 10.1073/pnas.1303897110 23776216 10.1073/pnas.1303897110 PMC3703987

[89] Lebeer S, Claes I, Tytgat HL, Verhoeven TL, Marien E, et al (2012) Functional analysis of *Lactobacillus rhamnosus* GG pili in relation to adhesion and immunomodulatory interactions with intestinal epithelial cells. Appl Environ Microbiol 78: 185–193. doi: 10.1128/AEM.06192-11 22020518 10.1128/AEM.06192-11 PMC3255643

[90] Hynonen U, Palva A (2013) *Lactobacillus* surface layer proteins: structure, function and applications. Appl Microbiol Biotechnol 97: 5225–5243. doi: 10.1007/s00253-013-4962-2 23677442 10.1007/s00253-013-4962-2 PMC3666127

[91] Johnson BR, O'Flaherty S, Goh YJ, Carroll I, Barrangou R, et al (2017) The S-layer associated serine protease homolog PrtX impacts cell surface-mediated microbe-host interactions of *Lactobacillus acidophilus* NCFM. Front Microbiol 8: 1185 doi: 10.3389/fmicb.2017.01185 28713337 10.3389/fmicb.2017.01185 PMC5491966

[92] Taverniti V, Stuknyte M, Minuzzo M, Arioli S, De Noni I, et al (2013) S-layer protein mediates the stimulatory effect of *Lactobacillus helveticus* MIMLh5 on innate immunity. Appl Environ Microbiol 79: 1221–1231. doi: 10.1128/AEM.03056-12 23220964 10.1128/AEM.03056-12 PMC3568609

[93] Mora D, Arioli S (2014) Microbial urease in health and disease. PLoS Pathog 10: e1004472 doi: 10.1371/journal.ppat.1004472 25501953 10.1371/journal.ppat.1004472 PMC4263730

[94] Millward DJ, Forrester T, Ah-Sing E, Yeboah N, Gibson N, et al (2000) The transfer of 15N from urea to lysine in the human infant. Br J Nutr 83: 505–512. 10953675

[95] Wilson CM, Loach D, Lawley B, Bell T, Sims IM, et al (2014) *Lactobacillus reuteri* 100–23 modulates urea hydrolysis in the murine stomach. Appl Environ Microbiol 80: 6104–6113. doi: 10.1128/AEM.01876-14 25063664 10.1128/AEM.01876-14 PMC4178674

[96] Power DA, Burton JP, Chilcott CN, Dawes PJ, Tagg JR (2008) Preliminary investigations of the colonisation of upper respiratory tract tissues of infants using a paediatric formulation of the oral probiotic Streptococcus salivarius K12. Eur J Clin Microbiol Infect Dis 27: 1261–1263. doi: 10.1007/s10096-008-0569-4 18560907 10.1007/s10096-008-0569-4

