# Correction: Comparative genomic analysis of the multispecies probiotic-marketed product VSL#3

**DOI:** 10.1371/journal.pone.0203548

**Published:** 2018-08-30

**Authors:** François P. Douillard, Diego Mora, Robyn T. Eijlander, Michiel Wels, Willem M. de Vos, The PLOS ONE Editors

The authors and the *PLOS ONE* Editors provide the following updates to this article [1].

The published Competing interests statement is incomplete and the relevant section should read: “Actial Farmaceutica SRL (formerly CDI Investments SRL) partly funded this study and provided the VSL#3 strains. This funder, like the other funders, had no role in study design, data collection and analysis, decision to publish, or preparation of the manuscript. In addition, we have included all information about the funders of this work and the employment of two of the co-authors in a small company NIZO. This does not alter our adherence to *PLOS ONE* policies on sharing data and materials.”

Regarding materials availability, the authors have clarified that enquiries regarding access to the bacterial strains should be directed to Actial Farmaceutica SRL via the following contact information: ACTIAL FARMACEUTICA SRL, Viale Shakespeare, 47, 00144, Rome–Italy; Phone: +39 0654211033, Fax: +39 065922448, Email: info@vsl3pharma.com

Questions were raised as to the referenced properties of VSL#3. All research conducted for this study used individual VSL#3 bacterial strains provided by Actial Farmaceutica SRL (Rome, Italy). The reported genome sequences and predicted functions of the VSL#3 strains form a basis for understanding the functionality of the VSL#3 product as well as conducting future studies on its stability, production or formulation.

The VSL#3 product was not included in these studies, as noted in the Methods. As such, the title and several statements throughout the article which refer to the VSL#3 product ought to refer to the bacterial strains rather than the product itself.

## References

[1] Douillard FP, Mora D, Eijlander RT, Wels M, de Vos WM (2018) Comparative genomic analysis of the multispecies probiotic-marketed product VSL#3. PLoS ONE 13(2): e0192452 10.1371/journal.pone.0192452 29451876 PMC5815585

