# Supplementary figures and images for: Comparative genomic analysis of the multispecies probiotic-marketed product VSL#3
Source: PLoS One. 2018 Feb 16;13(2):e0192452. doi: 10.1371/journal.pone.0192452 (PMC5815585; doi:10.1371/journal.pone.0192452)

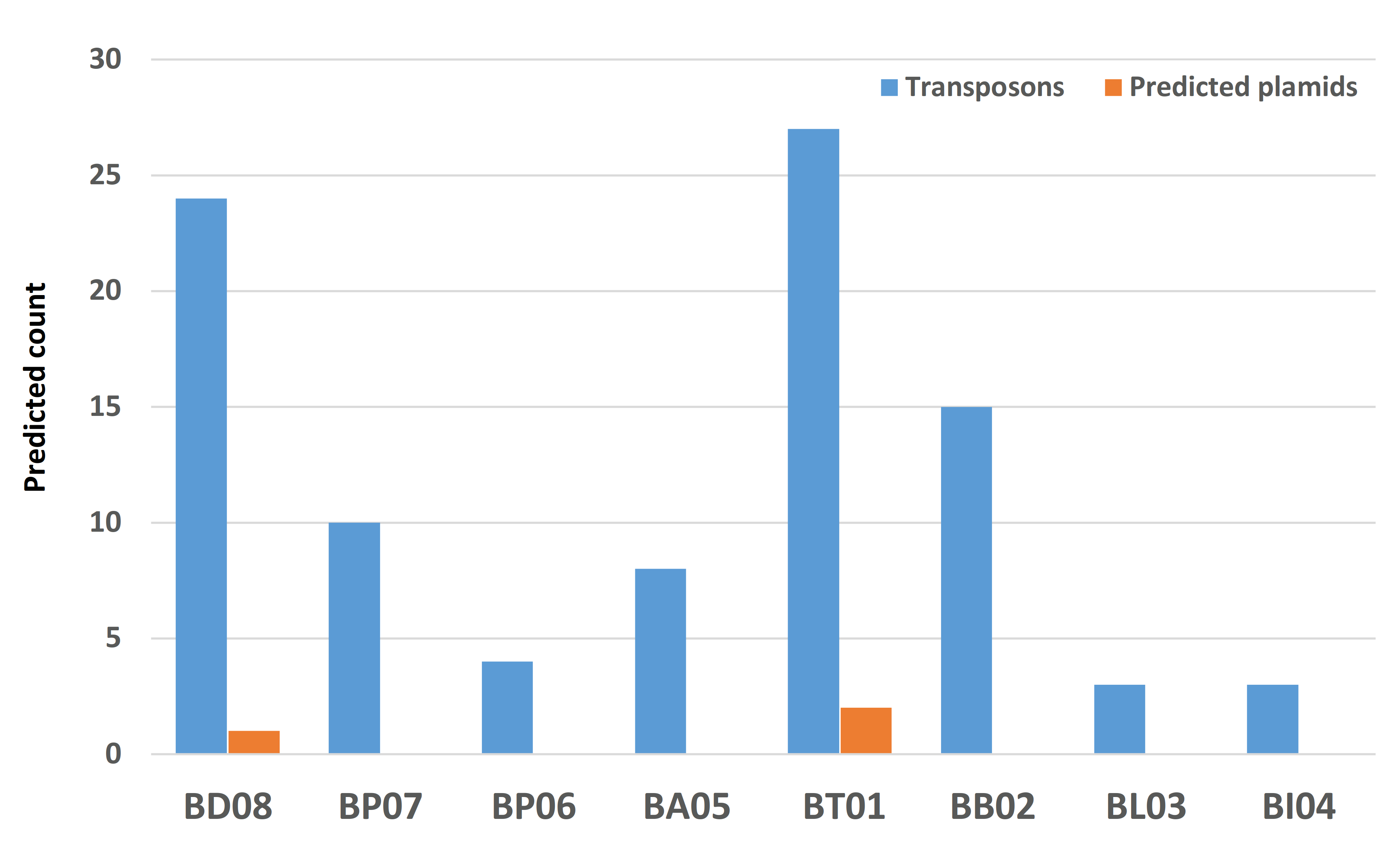

Supplement: S1 Fig — (TIF) [file pone.0192452.s001.tif]
