# Supplementary material for: Comparative genomic analysis of the multispecies probiotic-marketed product VSL#3
Source: PLoS One. 2018 Feb 16;13(2):e0192452. doi: 10.1371/journal.pone.0192452 (PMC5815585; doi:10.1371/journal.pone.0192452)
Supplement: S1 Table — (DOCX) [file pone.0192452.s002.docx]

**S1 Table. List of bacterial strains present in the multispecies probiotic-marketed product VSL#3 and growth conditions used in the present study.**

| Bacterial strain | Growth Medium | Temperature (°C) | Anaerobiosis | 24h-growth |
| --- | --- | --- | --- | --- |
| *Bifidobacterium breve* BB02  *Lactobacillus helveticus* BD08  *Lactobacillus paracasei* BP07  *Lactobacillus plantarum* BP06  *Streptococcus thermophilus* BT01  *Lactobacillus acidophilus* BA05  *Bifidobacterium animalis* subsp. *lactis* BL03  *Bifidobacterium animalis* subsp. *lactis* BI04 | MRS+ 0.05% cysteine  MRS  MRS  MRS  GM17  MRS  MRS+ 0.05% cysteine  MRS+ 0.05% cysteine | 37  37  37  37  37  37  37  37 | yes  no  no  no  no  yes/no  yes  yes | ++  +  ++  ++  ++  ++  ++  ++ |
