# Supplementary material for: Comparative genomic analysis of the multispecies probiotic-marketed product VSL#3
Source: PLoS One. 2018 Feb 16;13(2):e0192452. doi: 10.1371/journal.pone.0192452 (PMC5815585; doi:10.1371/journal.pone.0192452)
Supplement: S2 Table — (DOCX) [file pone.0192452.s003.docx]

**S2 Table. MIC of *Lactobacillus paracasei* LMG12586 against several antibiotics compared to reference values.** The MICs were determined in LSM (ISO-Sensitest broth, Oxoid supplemented with 10% v/v MRS Difco) as described [40] and recommended by EFSA [39]. * range of values reported in ISO10932/IDF223 2010 interlaboratory trial. n.d, not determined as not required by EFSA [39].

| Molecule | MIC (µg/ml) | ISO10932 (µg/ml)* |
| --- | --- | --- |
| ampicillin  vancomycin  gentamycin  kanamycin  streptomycin  erythromycin  clindamycin  tetracycline  chloramphenicol | 1  >16  2  64  16  0.25  0.125  2  8 | 0.5-2  n.d.  1-4  16-64  8.32  0.062-0.25  0.062-0.25  1-4  4-8 |
